# Motivated Shield From Chronic Noise Environment: Moderation of the Relationship Between Noise Sensitivity and Work Wellbeing by Need for Closure

**DOI:** 10.3389/fpsyg.2018.00663

**Published:** 2018-05-28

**Authors:** Stefano Livi, Gennaro Pica, Giuseppe Carrus, Marika Rullo, Marta Gentile

**Affiliations:** ^1^Department of Social and Developmental Psychology, Sapienza Università di Roma, Rome, Italy; ^2^Dipartimento di Scienze della Formazione, Università degli Studi Roma Tre, Rome, Italy; ^3^School of Psychology, University of Kent, Kent, United Kingdom

**Keywords:** need for cognitive closure, cognitive energetics theory, environmental noise, noise sensitivity, wellbeing, workplace

## Abstract

Several studies have underlined how chronic exposure to environmental noise may have negative effects on performance, wellbeing, and social relations. The present study (*N* = 90 employees of a motor factory who are chronically exposed to environmental noise) investigated whether the negative effects of chronic exposure of noise-sensitive individuals to noise in the workplace may be moderated by the need for cognitive closure (i.e., an epistemic tendency to reduce uncertainty; NFCC, Kruglanski, 2004). As NFCC has been shown to enhance protection of the focal goal by reducing interference (Kossowska, 2007; Pica et al., 2013), we hypothesized that people with high NFCC should be able to manage the interference coming from environmental noise and concentrate on their tasks more than their low-NFCC counterparts. The results confirm that the negative effects of noise sensitivity on job satisfaction, state anxiety, and turnover intent were higher among low (vs. high)-NFCC individuals.

## Introduction

Environmental noise is one of the major health risk factors in our current society, not only for its potential damages to aural function (hearing impairment) but also for its adverse impacts on neurological, psychological, and physiological systems (Evans, 2001; Ising and Kruppa, 2004). Specific categories of people such as industrial workers seem to be more affected by the negative health consequences of noise exposure (Mahendra Prashanth and Sridhar, 2008). The present research aims to investigate the motivational factors that may potentially buffer the negative effects of exposure to chronic noise. We explored the possibility that workers under chronic noise conditions may need greater than average capacity for focusing on the task at hand to overcome such interference and guarantee task execution. As we will introduce later, the *need for cognitive closure* (NFCC; i.e., desire for clarity in knowledge and aversion toward epistemic uncertainty; Kruglanski, 2004) is a motivational factor that captures such tendencies. In what follows, we first review the theoretical framework of the present research and then we present our hypotheses.

### Environmental Noise

Exposure to occupational noise leads to a variety of negative effects on physical and psychological wellbeing (Leather et al., 2003), as evidenced by findings of increased motivational deficits, musculoskeletal disorders (Evans and Stecker, 2004), and sickness-related absenteeism (Cohen, 1973) among those exposed to high noise levels. Consistent with this reasoning, research suggests that chronic noise can cause annoyance and mental fatigue (e.g., Lipscomb and Roettger, 1976); stress, physical fatigue, and post-work irritability (Melamed and Bruhis, 1996), psychological distress (McDonald, 1989); and a reduction of job satisfaction and performance (Sundstrom et al., 1994; for a review about the negative effects of noise on health, see Olaosun et al., 2009).

However, the effects of noise on performance are not clear-cut, i.e., some studies have found that performance decreases in a noisy environment while other studies have shown that noise does not affect performance (Auble and Britton, 1958). This last result is evidently due to individual differences in how people react to noise; some may be seriously affected while others not affected at all. Thus, an important issue for research in this area is an examination of individual difference factors that may reduce the negative effects of noise and at least in part explain the inconsistent results in studies of the relationship between noise and performance (Belojevic et al., 2003).

In this vein, individual differences in noise sensitivity have been suggested to be a critical factor in determining the magnitude of negative effects of chronic noise exposure (Taylor, 1984; Job, 1999). The concept of noise sensitivity captures the person’s attitudes toward environmental noise (Zimmer and Ellermeier, 1999). Individuals with strong noise sensitivity have been shown to be more likely to express annoyance and anger about irritating situations (Weinstein, 1978), to suffer greater levels of anxiety (Zimmer and Ellermeier, 1999), and to be characterized by lower intellectual ability, fewer social skills, and a stronger desire for privacy (Weinstein, 1978) than individuals who are less sensitive. Consistently, noise sensitivity has been found to be positively correlated with neuroticism (Ramirez et al., 2004) and physiological stress (Waye et al., 2002).

Although it can be assumed that noise sensitivity generally leads to poorer coping with environmental noise (i.e., reduced satisfaction, higher anxiety, and stress) during task performance, some research has not found a relationship between noise sensitivity and wellbeing (e.g., Bhatia et al., 1996).

Thus, it is important to examine what factors may moderate the negative effects of noise sensitivity. We argue that the negative effects of exposure to chronic noise among noise-sensitive workers may be moderated by factors that help isolate the self from interference, an ability captured in the concept of NFCC (Kruglanski, 2004). In the next section, we introduce the NFCC construct in more detail.

### Need for Cognitive Closure

Need for cognitive closure is defined as a desire for a definite answer to a question, any firm answer, rather than uncertainty, confusion, and ambiguity (Kruglanski, 2004; Roets et al., 2015). Individuals’ NFCC can vary stably across individuals and also across situations: levels of NFCC are increased in circumstances wherein cognitive processing is more difficult or unpleasant (e.g., noise, fatigue, or time pressure).

Individuals with a strong need for closure tend to “seize” on information, allowing for a quick judgment on a topic of interest and to “freeze” on such judgment, becoming relatively impermeable or closed minded to further relevant information (Kruglanski and Webster, 1996). Individuals with a strong need to avoid closure, by contrast, tend to keep information processing open, thus eschewing binding and definite points of view.

Even though recent findings sustain that NFCC—under conditions where lengthier and more effortful means are instrumental to attain closure—may actuate more systematic cognitive processing (Jaśko et al., 2015; Roets et al., 2015; Kossowska et al., 2016; Strojny et al., 2016; Szumowska and Kossowska, 2017), in general, previous research has shown that NFCC more often leads to less effortful, faster, and more superficial information processing (e.g., they base judgments on early appearing information, preexisting attitudes, and stereotypes; see Kruglanski and Webster, 1996; for overview). The above findings indicate that high-NFCC individuals strive for simplification, order, and predictability in order to reduce uncertainty and reach epistemic clarity. Consistent with this view, NFCC has been shown to enhance sensitivity to social environments and group norms as epistemic providers (Kosic et al., 2014; Gelfand and Harrington, 2015; Livi et al., 2015a,b; Pierro et al., 2015).

Importantly for the present research, NFCC has recently been shown to enhance goal shielding (i.e., protecting the focal goal with the inhibition of alternative goals; Shah et al., 2002), and to enhance focus on relevant information from the environment while inhibiting interference from irrelevant information (Kossowska, 2007; Pica et al., 2014). Even more recently, evidence has further corroborated this hypothesis by showing that NFCC enhanced multitasking performance due to better attentional selectivity and focus on the main task goal (Szumowska and Kossowska, 2017). The above tendency enhanced by high NFCC is achieved by banning (or inhibiting) from conscious awareness interfering materials, and it is particularly important when interference is high (Pica et al., 2013, 2014).

Moving from these findings, we assumed that in conditions where focus on one’s activities is undermined, namely in distracting conditions such as the presence of environmental noise, uncertainty, and confusion might be increased. In such conditions, higher levels of NFCC should help people to isolate themselves from distractions, thus protecting focal activities from uncertainty and confusion created by their presence. Hence, we propose that high NFCC should buffer the negative effects induced by chronic noise because high-NFCC individuals might be more able to cope with the disturbing environment; by contrast, individuals with low NFCC should suffer more from the negative effects of working in a chronically noisy environment because of their lower abilities to manage interference.

### The Present Research

The present study aims to assess whether the negative effects induced by chronic exposure to environmental noise among noise-sensitive individuals can be moderated by individual differences in NFCC. More specifically, we hypothesize that for noise-sensitive individuals, high levels of NFCC should buffer the negative effects of exposure to noise by more successfully managing its interference with work activities. On the other hand, noise-sensitive individuals with comparatively low levels of NFCC should suffer greater negative consequences from exposure to noise because it is a greater hindrance to focusing on their work activities. The above idea is directly driven by the need for closure theory, according to which, to arrive at a clear-cut conclusion and avoid uncertainty that may derive from the presence of disturbances (such as noisy environments during task execution), NFCC isolates the individual from interference by allowing her/him to focus on her/his activities (see Shah et al., 2002; Kossowska, 2007; Pica et al., 2014; Szumowska and Kossowska, 2017). This should be especially true for individuals showing higher levels of sensitivity to noise, as we assume the interference to be stronger for such individuals given that they show more negative reactions (such as annoyance and dissatisfaction) to noise (see Job, 1999).

Notably, although there has been ample research on the effects of occupational noise, as we have seen before, it has mainly focused on office environments, while few studies have investigated the effects of noise exposure in industrial settings, such as machine intensive production sites (Kryter, 1994). This is an important point, since a high percentage of workers in industry are exposed to high levels of noise (above 85 dB), often chronically, throughout their workday (Raffaello and Maas, 2002). Therefore, in the present study we choose to test our hypotheses in the context of an industrial setting.

## Materials and Methods

### Participants

A total of 90 employees of a motor factory who are exposed to environmental noise were recruited for the present study and administered the questionnaires in two different departments: warehouse and picking. The mean dbA of personal daily exposition (L_PE.d_) was equal to 75.1. Employees were exposed to the noise 418 min per day^1^. According to the current regulatory standards in Europe mentioned before, these levels are in conformity to the law. However, because this value is relatively close to the legal lower exposure action value, we can argue that our participants are exposed to chronic workplace noise, at levels that we might assume as having potentially dangerous outcomes for neurological, psychological, and physiological systems (Directive 2003/10/EC of the European Parliament and of the Council). In sum: *N* = 90; *N* = 66 for male workers and *N* = 24 for female workers. Mean age was 34.9 years (*SD* = 6.7; range 21–53). Five participants (5.5%) had college degrees, seventy-one (78.9%) were high school graduates, and fourteen (15.6%) had a junior high school education. We performed *post hoc* analysis for the estimation of the statistical power of our study using G^∗^Power 3.1.9.2. One-tailed power analysis with an α error of 0.05 revealed a statistical power of 93% for our sample size.

### Procedure and Measures

Participants were asked to complete an anonymous, paper-and-pencil self-administered questionnaire composed of:

(1) A shortened version of the *Revised NFCC Scale* (Pierro and Kruglanski, 2005, Unpublished), measuring the dispositional tendency of need for closure, and consisting of 14 items (α = 0.71) each rated on a 5-point scale ranging from strongly disagree to strongly agree (e.g., “Any solution to a problem is better than remaining in a state of uncertainty”). Previous studies have demonstrated that the shortened version of the NFCC has nomological validity (the disattenuated correlations between Rev NFCC and the previous version of the NFCC in United States and Italian samples are 0.92 and 0.93, respectively) and satisfactory reliability (see Pierro et al., 2015).
(2) The *Weinstein Noise Sensitivity Scale* (Weinstein, 1978; Senese et al., 2012), measuring the subjective level of noise sensitivity, and consisting of 21 items (α = 0.88) each rated on a 5-point scale ranging from strongly disagree to strongly agree;
(3) The *STAI-State-Trait Anxiety Inventory* – *State Version* (Spielberger et al., 1980), measuring the self-reported levels of anxiety of the individual, consisting of 20 items (α = 0.89). To complete the scale, participants color in a numbered circle (1 = not at all; 2 = somewhat; 3 = moderately so; 4 = very much so) to indicate their current state for each statement;
(4) The *Job Satisfaction Scale*, measuring job satisfaction with four items (α = 0.71) derived from Brayfield and Rothe’s (1951) job satisfaction scale, each rated on a 5-point scale ranging from strongly disagree to strongly agree (e.g., “Most days I am enthusiastic about my work”).
(5) A composite turnover intention score was assessed through three items from Mobley’s (1977) turnover intention measure (e.g., “I have often seriously considered finding a job elsewhere”). Participants’ responses were recorded on a 6-point scale ranging from 1 (strongly disagree) to 6 (strongly agree) (α = 0.87).

A section measuring participants’ socio-demographic characteristics was also included.

### Statistical Analyses

A moderated regression model was used, presuming that the relationship between noise sensitivity and the job wellbeing variables are altered linearly by NFCC (Baron and Kenny, 1986; Kenny, 2010) (see **Table 1** for descriptives statistics and correlation matrix).

**Table 1:** Descriptive statistics and zero-order correlations among variables.

Variable	Mean	*SD*	1	2	3	4	5
(1) Noise sensitivity	3.11	0.79	-				
(2) Need for cognitive closure	3.34	0.54	0.24^∗^	-			
(3) Job satisfaction	3.68	1.00	-0.13	0.16	-		
(4) STAI	1.87	0.48	0.23	-0.08	-0.33^∗∗^	-	
(5) Turnover intent	2.46	1.25	0.07	-0.09	-0.47^∗∗^	0.50^∗∗^	-

*^∗^p < 0.05; ^∗∗^p < 0.01.*

In our analysis, the predictor variable was noise sensitivity, the outcome variables were STAI, job satisfaction and turnover intent (DVs), and the moderator variable was NFCC. The model is as follows: The variable noise sensitivity is presumed to cause the DVs linearly, and its effect is presumed to be altered linearly by NFCC. Because zero is not a possible value for either noise sensitivity or NFCC, both these variables were grand-mean centered before the moderation analysis.

## Results

**FIGURE 1:**
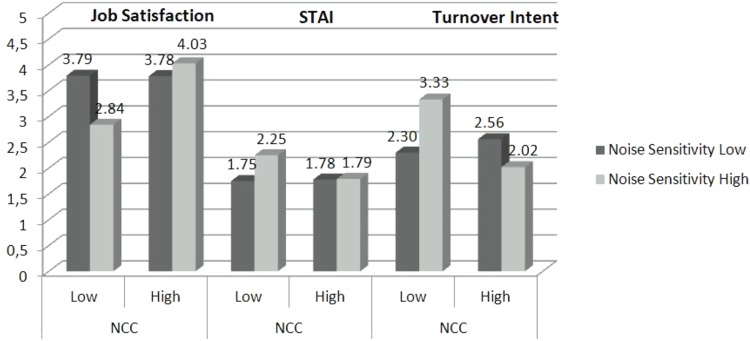
Predicted means for noise sensitivity (–1 and +1 SD) and need for cognitive closure (–1 and +1 SD) for each outcome variable. GHQ, general health questionnaire; STAI, state-trait anxiety’ inventory.

The results of the moderated regression analysis are summarized in **Table 2**: the results of the moderated regression analysis for job satisfaction are similar but in the opposite direction (Model 1). The overall effect of noise sensitivity on job satisfaction is -0.229 and marginally significant (*p* = 0.090) (see **Table 2** and **Figure 1**). The effect of NFCC is significant (0.553; *p* = 0.010). In the case of job satisfaction, the interaction between noise sensitivity and NFCC is statistically significant and equal to 0.710 (*p* = 0.029). As the NFCC increases, the effect of noise sensitivity is weakened proving again that a null relationship occurs when NFCC is high. The standardized effect of noise sensitivity for people who are one standard deviation below the mean of NFCC is equal to -0.610 and significant (*p* = 0.006) while the effect of noise sensitivity for those who are one standard deviation above the mean of NFCC is non-significant (*b* = 0.153; *p* = 0.483).

**Table 2:** Results of moderation analysis with noise sensitivity, need for closure, and the three outcome variables.

Predictor	Model 1: job satisfaction			Model 2: STAI			Model 3: turnover intent		
	Estimate	SE	*p*	Estimate	SE	*p*	Estimate	SE	*p*
Intercept	3.611	0.106	<0.001	1.894	0.050	<0.001	2.555	0.130	<0.001
Noise sensitivity	-0.229	0.133	0.090	0.162	0.063	0.012	0.155	0.163	0.346
NFCC	0.553	0.211	0.010	-0.199	0.100	0.051	-0.490	0.258	0.061
NFCC^∗^NS	0.710	0.319	0.029	-0.286	0.152	0.062	-0.932	0.391	0.019
Model summary	*R*^2^ = 0.11 (*p* = 0.017)			*R*^2^ = 0.11 (*p* = 0.020)			*R*^2^ = 0.08 (*p* = 0.069)		

*NFCC, need for cognitive closure; NS, noise sensitivity; STAI, state-trait anxiety inventory.*

The results of the moderation model when using STAI as the outcome variable are similar but in the opposite direction (Model 2): the overall effect of noise sensitivity is 0.162 (*p* = 0.012). The overall effect of NFCC is -0.199 (*p* = 0.051). The interaction between noise sensitivity and NFCC is equal to -0.286 and is marginally statistically significant (*p* = 0.062). Nevertheless the simple slope analysis results are in the predicted direction: the standardized effect of noise sensitivity for persons who are one standard deviation below the mean on NFCC is significant and negative (*b* = -0.316; *p* = 0.003), while the effect of noise sensitivity for persons who are one standard deviation above the mean on NFCC is non-significant, equal to 0.008 (*p* = 0.938).

Finally, the results of the moderation model when using turnover intent as the outcome variable are similar to those emerged for STAI (Model 3): the overall effect of noise sensitivity is 0.155 (*p* = 0.346) while the effect of NFCC is -0.490 (*p* = 0.061). Most importantly, the interaction between noise sensitivity and NFCC is equal to -0.932 and is statistically significant (*p* = 0.019). The simple slope analysis results goes in the same direction of STAI: the standardized effect of noise sensitivity for persons who are one standard deviation below the mean on NFCC is significant, equal to 0.655 (*p* = 0.016) while the effect of noise sensitivity for persons who are one standard deviation above the mean on NFCC is non-significant and negative (*b* = -0.346; *p* = 0.196).

## Discussion

The aim of the present research was to examine whether the negative effects of working in a chronic noisy environment experienced by high noise-sensitive workers (i.e., those with high anxiety and low job satisfaction) might be moderated by individuals’ tendency toward NFCC (Kruglanski, 2004).

Building on previous research on the NFCC (Shah et al., 2002; Kossowska, 2007; Pica et al., 2014; Szumowska and Kossowska, 2017), we conceptualized chronic environmental noise as an interfering force, hindering task execution at work, and NFCC as a factor helping people to protect their work execution from the interference of noise. We hypothesized that high noise-sensitive individuals with low levels of NFCC should suffer *more* negative effects of chronic noise, while their high-NFCC counterparts should *buffer* those effects; i.e., NFCC should enhance protection of task execution from the interference of noise and moderate the influence of noise on wellbeing and job satisfaction outcomes.

Consistent with our hypotheses, although (similarly to Mahendra Prashanth and Sridhar, 2008, and many others) we found negative relationships between noise sensitivity and job satisfaction, health and wellbeing among workers in an environment characterized by chronic noise exposure, the above relationships were moderated by individual differences on the NFCC. In particular, low-NFCC individuals with high noise sensitivity exhibited a higher level of negative consequences of exposure to noisy working environment than did their high-NFCC counterparts.

These results are consistent with previous work showing that high-NFCC individuals are more task oriented (Pierro et al., 2003), protect the focal goal by inhibiting alternative goals (Shah et al., 2002), and better remove potentially distracting cognitive elements (Kossowska, 2007; Pica et al., 2014; Szumowska and Kossowska, 2017), all characteristics that may help in handling the interference coming from noisy working conditions. In fact, the tendency of NFCC to focus on specific categories, or concepts, represents a mechanism allowing for higher cognitive selectivity and more success at shutting out irrelevant distractions such as noise. In addition, our results are also in line with previous studies in the work and organizational psychology field. For instance, a recent study by Chirumbolo and Areni (2010) showed that the effects of another stress source in the workplace (job insecurity) might be moderated by dispositional NFCC: low-NFCC individuals were more affected than their high-NFCC counterparts by the damaging health impacts of job insecurity. Of course, job insecurity and exposure to noisy working environment are different concepts; however, these two variables can both be considered as interferences with daily working activities and both produce similar detrimental effects on working wellbeing—and high NFCC helps to manage both of these sources of interference and protect individuals from their negative impact on wellbeing at work.

More generally, our findings are also consistent with the idea of a relationship between perception of environmental stimuli, cognitive processes, social behavior, and human wellbeing (e.g., Carrus et al., 2015, 2017; Mercado-Doménech et al., 2017). In particular, from a practical point of view, the findings of the present research contribute to the study of individual differences in managing the negative effects of chronic noisy environments on wellbeing at work. We identified two factors that may moderate such effects, namely noise sensitivity and NFCC. Individuals characterized by high dispositional noise sensitivity and low dispositional motivation toward closure suffer such effects more than those with low noise sensitivity, but noise-sensitive individuals who are high in NFCC are no more affected by noise than those with low noise sensitivity. Although there are individual differences in reacting to noisy environments, reduction of workers’ exposure to noise—wherever possible—must be a priority for enterprises. Furthermore, given that low-NFCC workers with high sensitivity to noise suffer more from the negative effects of noise, organizations should be aware that goals potentially inducing low NFCC tendencies in their workers, such as accountability or accuracy (Webster et al., 1996; Roets et al., 2015), may be counterproductive in the presence of interfering and disturbing factors such as chronic noise. Thus, managers may regulate levels of accountability and/or accuracy accordingly, in order to create the best possible conditions for workers subjected to chronic noise.

### Limitations and Future Directions

Some limitations of the present study should also be taken into consideration and be addressed in future research. First, our data set is limited to 90 participants, which limits the generalizability of our results. Moreover, with our design, we cannot actually draw causal inferences. Therefore, future studies using quasi-experimental or longitudinal research designs are needed to test the predicted causal paths and corroborate the findings of the present research. Second, in our sample no data were available for the estimation of workers’ actual hearing abilities. As hearing ability may moderate noise sensitivity (Senese et al., 2012) by enhancing or reducing the perception of noise and its negative outcomes, future studies should control for this variable. Last, future studies might also assess wellbeing outcomes using psychophysiological measures (e.g., blood pressure and pulsation). This might provide more direct evidence of the negative effects of chronic noise exposure among noise-sensitive individuals and the moderating role of NFCC.

## Conclusion

To conclude, the results we obtained show that among noise-sensitive individuals, those with a high-NFCC suffer less from the negative effects of chronic environmental noise than their low-NFCC counterparts. We argued that although environmental noise can interfere with task execution, a high level of NFCC helps to isolate the person from this interference, thus protecting task execution and job satisfaction and mitigating anxiety. The paper integrates literature on NFCC and research on noise environment and noise sensitivity, and pertains to the more general question of what factors moderate the negative effects of noisy circumstances at work.

## Ethics Statement

This study, conducted with self administered questionnaire, was carried out in accordance with the recommendations of Ethical Committee of the Department of Social and Developmental Psychology for the use of Human participants of Sapienza University of Rome and the protocol was approved 10/11/2015 (Prot 645; pos 111/14).

## Author Contributions

SL collected the data. SL and GP conducted the data analysis and interpreted the findings. SL, GP, MR, MG, and GC drafted the manuscript and provided critical revision of the manuscript for important intellectual content. All authors critically reviewed the content and approved the final version for publication.

## Conflict of Interest Statement

The authors declare that the research was conducted in the absence of any commercial or financial relationships that could be construed as a potential conflict of interest.

## Footnotes

1 Measurement of noise was obtained during periodic acoustic control (2 days) using Bruel and Kiaer (mod.2231–2236).
